# Bacteriology in Medicine and Surgery

**Published:** 1900-04

**Authors:** 


BACTERIOLOGY IN MEDICINE AND SURGERY. A Practical Manual for Physicians, Health Officers and Students. By WILLIAM H. PARK, M. D, Associate Professor of Bacteriology and Hygiene in the University of Bellevue Hospital Medical College, New York. In one 12mo volume of 688 pages, with 87 illustrations in black and colors, and two full-page colored plates. New York and Philadelphia: Lea Brothers & Co. 1900.

The author has attempted to bring together the facts concerning bacteriology which will constitute a text-book sufficient for the needs of the student and of direct practical value to the physician and health officer. Dr. Park is eminently qualified for the authorship of a work of this kind inasmuch as he has been assistant pathologist for many years in the research laboratories of the Department of Health in the City of New York. In this capacity he has done much for practical bacteriology and made his laboratory the Mecca for health officers and sanitarians, who are striving to keep in touch with the latest phases and practices of preventive medicine. In the work before us the author strives to place the essentials of bacteriology before the physician, leaving the carrying out of detail and more difficult examinations to the trained and experienced bacteriologist. This is a capital idea and one which might have been followed by other authors, as it must be patent even to the beginner that careful and therefore honest bacterial research can only be made in thoroughly equipped and up-to-date laboratories. The author particularly emphasises such subjects as the chemical changes produced by bacteria, infection, immunity, the nature and use of protective serums, and the diagnostic value of bacteriological cultures, since knowledge of these subjects is of special importance to the practising physician in that it enables him to obtain an intelligent grasp of the nature of the infectious diseases.

The methods used in the laboratory of the board of health in New York for the isolation and identification of the typhoid, tubercle, and diphtheria bacilli are given with especial fulness, as bacteriological examination of the discharges of persons suspected to have typhoid fever, tuberculosis, or diphtheria are now generally made for these bacteria in the laboratories of the health departments of even the smaller cities, because of the manifest importance to the public of knowing where such sources of infection exist.

Several very important and interesting chapters in the body of the book treat of such practical subjects as practical disinfection and sterilisation (house, person, instruments and food), sterilisation of milk for feeding infants. The various chemical agents are given—that have proved to be of especial worth; the formalin methods are given with much detail, the author having been a path-maker in the application and perfection of formalin methods. The chapter on water is timely, and the directions given for treating water are safe and sound.

In the appendix are given brief descriptions of a few representative pathogenic microörganisms which are not bacteria, such as actinomyces, plasmodium malariæ, ameba coli, the microörganism of smallpox, cowpox and rabies. The illustrations are all well chosen and nicely executed. Several full page colored plates help materially to embellish the work besides adding to its worth and usefulness. W. C. K.

